# Thickness of the cerebral cortex shows positive association with blood levels of triacylglycerols carrying 18-carbon fatty acids

**DOI:** 10.1038/s42003-020-01189-5

**Published:** 2020-08-20

**Authors:** Eeva Sliz, Jean Shin, Catriona Syme, Sandra Black, Sudha Seshadri, Tomas Paus, Zdenka Pausova

**Affiliations:** 1 The Hospital for Sick Children, University of Toronto, Toronto, ON Canada; 2 Departments of Physiology and Nutritional Sciences, University of Toronto, Toronto, ON Canada; 3 Department of Medicine (Neurology), University of Toronto, Toronto, ON Canada; 4 Toronto Dementia Research Alliance, Toronto, ON Canada; 5 Sunnybrook Research Institute, Toronto, ON Canada; 6 Sunnybrook Health Sciences Centre, Toronto, ON Canada; 7 Hurvitz Brain Sciences Program, Sunnybrook Health Sciences Centre, Toronto, ON Canada; 8 LC Campbell Cognitive Neurology Research Unit, Toronto, ON Canada; 9 The Framingham Heart Study, Framingham, MA USA; 10 Department of Neurology, Boston University School of Medicine, Boston, MA USA; 11 Bloorview Research Institute, Holland Bloorview Kids Rehabilitation Hospital, Toronto, ON Canada; 12 Departments of Psychology and Psychiatry, University of Toronto, Toronto, ON Canada

**Keywords:** Metabolomics, Neuroscience

## Abstract

Perturbations in fatty acid (FA) metabolism as well as thinning of the cerebral cortex have been associated with cognitive decline in the elderly. Predominant FAs in the brain are docosahexaenoic acid (DHA) and arachidonic acid (ARA). Approximately 2–8% of esterified DHA and 3–5% of esterified ARA in the brain are replaced daily. DHA and ARA are derivatives of 18-carbon essential FAs, α-linolenic acid and linoleic acid, that must be imported into the brain from the circulation. In blood, FAs are primarily transported in triacylglycerols (TAGs) from which they can be released at the blood–brain-barrier and transported inside the brain. We show that circulating levels of TAGs carrying 18-carbon FAs are positively associated with cortical thickness in middle-aged adults. These associations are stronger in cortical regions with higher expression of genes regulating long-chain FA metabolism and cellular membranes, and cortical thickness in the same regions may be related to cognitive performance.

## Abstract (web summary)

Eeva Sliz et al. show an association between triacylglycerols species carrying 18-carbon unsaturated fatty acids and cortical thickness by combining serum lipidomics and brain imaging data. This association is more pronounced in cortical regions with higher expression of genes regulating fatty acid metabolism and cellular membranes.

## Introduction

Fatty acids (FAs) are essential building blocks of other lipids, such as triacylglycerols (TAGs), phospholipids, and cholesteryl esters. The brain requires high quantities of FAs for normal structure and function. Docosahexaenoic acid (DHA; FA22:6ω3) and arachidonic acid (ARA; FA20:4ω6) are the predominant FAs in the brain, constituting ~20% of the dry weight of the human brain^1^. It has been estimated that 2–8% of esterified DHA and 3–5% of esterified ARA in the brain are replaced daily^2^. DHA and ARA are derivatives of α-linolenic acid (ALA, FA18:3ω3) and linoleic acid (LA, FA18:2ω6), respectively. These essential FAs, along with other polyunsaturated FAs (PUFAs), must be imported into the brain from the circulation, whereas saturated and monounsaturated FAs can be synthesized within the brain. In blood many of the circulating FAs are transported within TAGs that are synthesized in the liver and transported within lipoprotein particles. Lipoprotein particles can enter endothelial cells of the blood–brain-barrier (BBB) by endocytosis, and, subsequently, FAs can be released from TAGs^3^. Alternatively, lipoprotein particles can interact with membrane-bound lipoprotein lipase to release unesterified FAs that can enter BBB by transporter proteins or passive diffusion^3^. Eventually, TAG-derived FAs within endothelial cells can be transported into neuronal and non-neuronal cells of the brain to be used for phospholipid synthesis or other functions^3^.

FAs vary in the degree of saturation and length of their acyl-carbon chain. Generally, shorter-chain, saturated FAs are associated with higher risk of cardiovascular diseases, whereas longer-chain PUFAs have beneficial cardiovascular effects^4,5^. Higher dietary intake and circulating concentrations of PUFAs have been associated with better cognitive functioning and lower risk of neurodegenerative diseases^6–8^. The brain uses FAs mostly for synthesis of phospholipids, which are essential components of cell membranes^9^. PUFA derivatives have additional functions in the brain—they act as drivers of multiple signaling processes, such as inflammatory or endocannabinoid signaling^3^. Lack of PUFAs has a detrimental effect on normal brain physiology, arising from, among others, altered structural features of cellular membranes and defects in preventing neuroinflammation^6^.

In the present study, we show that circulating concentrations of particular TAG species carrying 18-carbon FAs, including the essential ALA and LA, associate with thicker cerebral cortex in middle-aged adults. We further demonstrate that these TAG species show larger effect sizes in cortical regions with higher expression of genes regulating long-chain FA metabolism and cellular membranes, and that cortical thickness in these regions may be related to cognitive performance.

## Results

Recent developments in lipidomics technologies have enabled identification of lipids with improved specificity and sensitivity. Here, we study the associations between cortical thickness and circulating concentrations of 518 TAG species (Supplementary Fig. 1 and Supplementary Data 1), each of which was characterized by the total number of acyl-chain carbons and double bonds and by the identity of one of the three esterified FAs. After excluding individuals on lipid-lowering medication, our study sample consisted of 441 middle-aged adults from the Saguenay Youth Study (SYS)^10^ in whom brain magnetic resonance images of the brain (1.5T Avanto, Siemens) were acquired and TAG species concentrations were quantified in fasted serum samples (TrueMass Complex Lipid Panel^TM^, Metabolon) were available. The basic characteristics of the studied participants are in Table 1.

**Table 1:** Study population characteristics.

Characteristic	Value^a^
Number	441
Males (%)	189 (43)
Age, years	49.1 ± 4.9
BMI, kg/m^2^	27.6 ± 5.2
Total TAG, mmol/L	1.21 ± 0.7
Mean cortical thickness, mm	2.43 ± 0.09
Total cortical surface area, m^2^	0.17 ± 0.016

*BMI* body mass index, *TAG* triacylglycerol.

^a^Values are mean ± standard deviation.

### TAG associations with cortical thickness

In linear regression models adjusted for age and sex, we found positive associations (*p* < 0.006) between mean cortical thickness and concentrations of 7 of 518 tested TAG species (TAG52:6FA18:3, TAG54:6FA18:3, TAG54:7FA18:1, TAG54:7FA18:3, TAG54:8FA18:2, TAG54:8FA18:3, TAG56:9FA18:3; Fig. 1 and Supplementary Data 1). Statistical significance was considered at *p* < 0.006 (0.05/9) to correct for the nine principal components explaining 95% of variation in serum concentrations of 518 TAG species^11–13^.

**Fig. 1:**
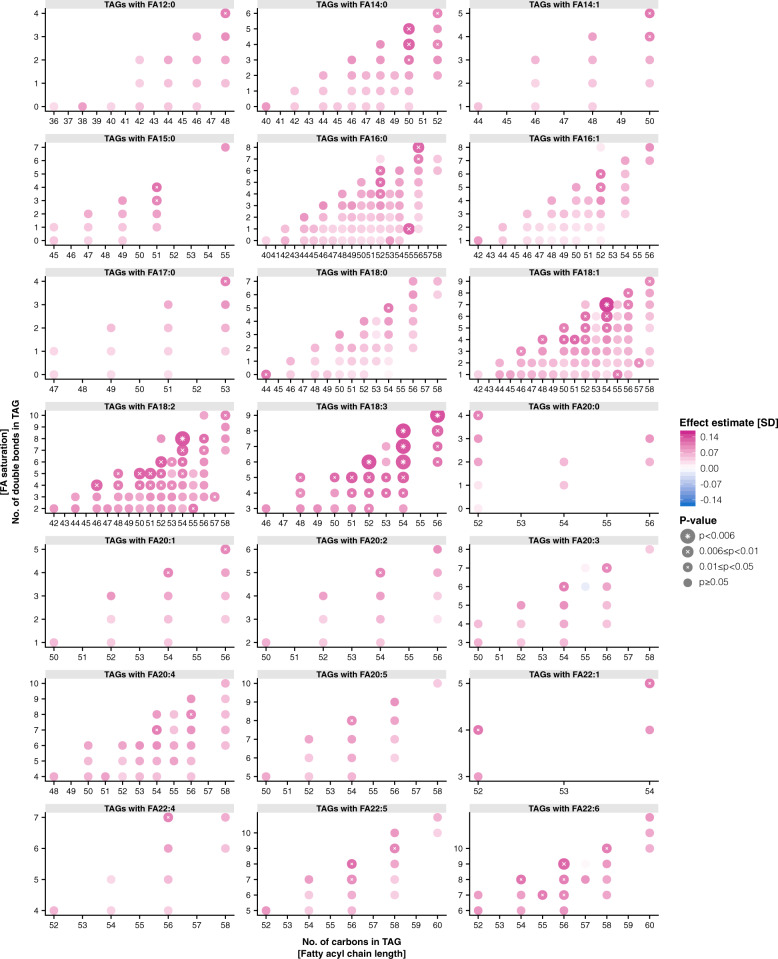
Associations between mean thickness of the cerebral cortex and serum concentrations of 518 triacylglycerols. We tested associations between mean thickness of the cerebral cortex and serum concentrations of 518 triacylglycerol species in middle-aged adults (*n* = 441) using linear regression models. Prior to model fitting, each variable was adjusted for age and sex, and transformed using rank-based inverse normal transformation. Statistical significance was considered at *p* < 0.006 (0.05/9) to correct for the first nine principal components explaining 95% of variation in serum concentrations of 518 triacylglycerol species. Each plot shows the results of the association tests by sets of triacylglycerol species carrying the same fatty acyl-chain; individual points indicate effect estimates (color) and *p*-values (size) for each of the triacylglycerol species. Triacylglycerol species are characterized by the total number of acyl-chain carbons (*x*-axes) and double bonds (*y*-axes) per triacylglycerol molecule. FA fatty acid, TAG,triacylglycerol; SD standard deviation.

**Fig. 2:**
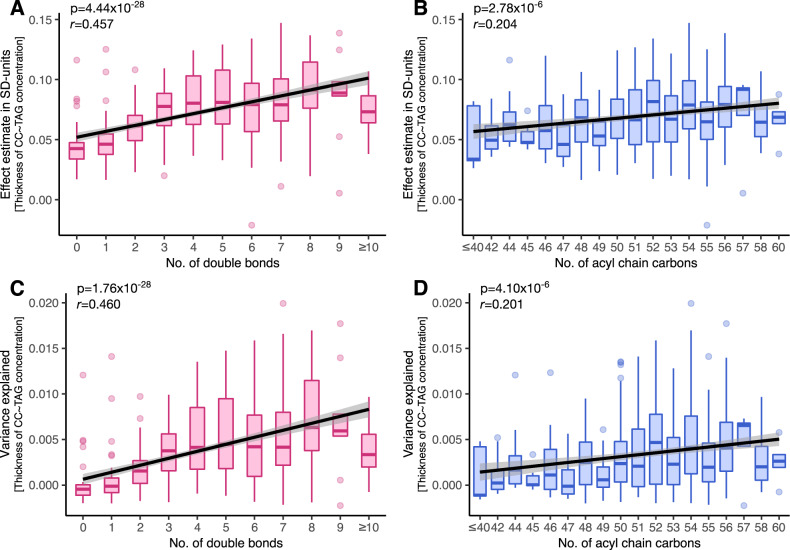
Correlations of fatty acyl unsaturation and chain length with the test statistics of the associations between mean thickness of the cerebral cortex and concentrations of 518 triacylglycerols. We studied if the association statistics of the mean thickness of the cerebral cortex with concentrations of 518 triacylglycerol species vary with fatty acyl unsaturation or chain length. To do this, we extracted effect estimates (*y*-axes in **a** and **b**) and coefficients of determination (adjusted *R*^2^; *y*-axes in **c** and **d**) from each of the 518 linear models, and studied their correlation (Pearson’s *r*) with the number of double bonds (*x*-axes in **a** and **c**) or the number of acyl-chain carbons (*x*-axes in **b** and **d**) per triacylglycerol species. CC cerebral cortex, SD standard deviation, TAG triacylglycerol.

Further, we found that the number of double bonds and the number of acyl-chain carbons per TAG species correlated positively with both the effect sizes obtained from the 518 regression models (*p* = 4.4 × 10^–28^ and *p* = 2.8 × 10^–6^, respectively) and the proportions of the variance in mean cortical thickness explained by these models (*p* = 1.8 × 10^–28^ and *p* = 4.1 × 10^–6^, respectively; Fig. 2). These findings indicate that the longer and more unsaturated the FAs carried by TAG molecules are, the stronger the positive associations between the concentration of those TAG species and cortical thickness are.

To evaluate if FAs per se drive these associations, we tested associations between mean cortical thickness and 21 FA species extracted from TAG fraction of lipids (TAG-FAs). Only TAG-FA18:3 (*p* = 0.0071), TAG-FA22:1 (*p* = 0.034), and TAG-FA22:6 (*p* = 0.036) showed nominally significant associations (Supplementary Fig. 2). We also tested associations between mean cortical thickness and total concentrations of 28 FA species (total-FA; from all circulating protein or lipid carriers, including TAGs). Only total-FA22:6 (*p* = 0.009) and total-FA24:0 (*p* = 0.043) showed nominally significant associations (Supplementary Fig. 3).

The results of supplemental analyses further adjusted for apolipoprotein E (*APOE*) alleles were comparable to the original findings (Supplementary Data 1 and Supplementary Fig. 4). We also tested the associations between total cortical surface area and TAGs, but we did not observe statistically significant associations (Supplementary Fig. 5).

### In silico analyses of TAG effect sizes across cortex

**Fig. 3:**
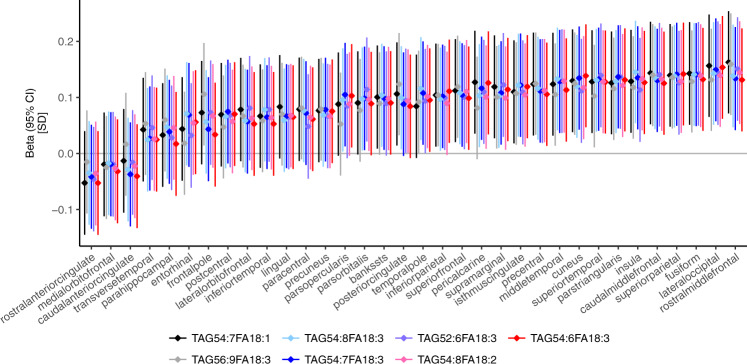
Associations of seven triacylglycerol species with cortical thickness in 34 regions of the cerebral cortex. Cortical thicknesses in each of the 34 regions (average over two hemispheres) were regressed on serum concentrations of each of the seven triacylglycerol species. Regression model fitting was done in a sample of 441 middle-aged adults. Before model fitting, all variables were adjusted for age and sex and inverse-rank normal transformed. TAG triacylglycerol, FA fatty acid, SD standard deviation, CI confidence interval.

We observed that the seven TAGs significantly associated with mean cortical thickness were associated similarly with thickness at each of the 34 regions of the cerebral cortex, as segmented by FreeSurfer using the Desikan-Killiany atlas^14^ (Fig. 3). The associations between seven TAGs and cortical thickness varied across the 34 regions, being most pronounced in the rostral middle frontal cortex and least pronounced in the anterior cingulate cortex (Fig. 3). From these region-specific association tests, we averaged effect estimates (betas) of the seven TAGs in each of the 34 regions, deriving an “interregional average-beta profile” (Supplementary Data 2). We utilized this “interregional average-beta profile” in downstream analyses to estimate (i) relative contributions of different cell types (virtual histology) and (ii) the cognitive implications.

In virtual histology analyses^15^, we found that the “interregional average-beta profile” was associated with interregional profiles of messenger RNA (mRNA) expression of genes specific to astrocytes (*p* = 1.9 × 10^–3^), CA1 pyramidal neurons (*p* = 1.9 × 10^–3^) and S1 pyramidal neurons (*p* = 3.0 × 10^–3^; Supplementary Fig. 6). These results suggest that the TAG associations with cortical thickness may involve these cell types. In functional element enrichment analyses, we found that astrocyte-specific genes were enriched in biological functions relevant to long-chain FA metabolic processes (13 genes with *r* > 0; Supplementary Data 3). Neuronal cell-specific genes were enriched in biological functions relevant to cell membrane, ion transport and actin remodeling (33 and 45 genes for CA1 and S1, respectively, with *r* > 0; Supplementary Data 4 and 5).

**Fig. 4:**
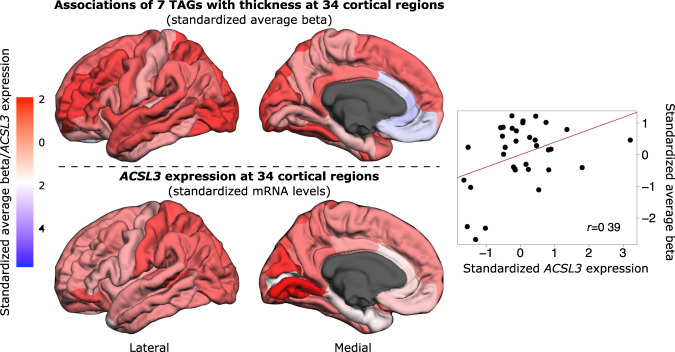
Interregional variations in the associations of seven triacylglycerols with cortical thickness and expression of ACSL3. Thirty-four cortical regions segmented by FreeSurfer are shown with color-coded values of the associations between seven triacylglycerols and cortical thickness (standardized average-beta) and *ACSL3* expression (standardized mRNA levels). Correlation between interregional variations in the average-beta of the seven triacylglycerols on cortical thickness and *ACSL3* expression is shown on the right. TAG triacylglycerol, *ACSL3* acyl-CoA synthetase long-chain family member 3.

Of note, the astrocyte-specific genes relevant to long-chain FA metabolic processes included *ACSL3*, encoding acyl-CoA synthetase long-chain family member 3, which is an enzyme required for the incorporation of long-chain FAs into cellular lipids^16^. ACSL3 is one of two predominant isoforms of this enzyme in the brain^17^. In the present study, the “interregional average-beta profile”, that was derived from the associations between seven TAGs and cortical thickness, correlated positively with the interregional profile of *ACSL3* expression (Fig. 4), indicating that the associations of seven TAGs with cortical thickness are more pronounced in cortical regions with higher expression of this gene.

### Correlations with cognitive functioning

**Fig. 5:**
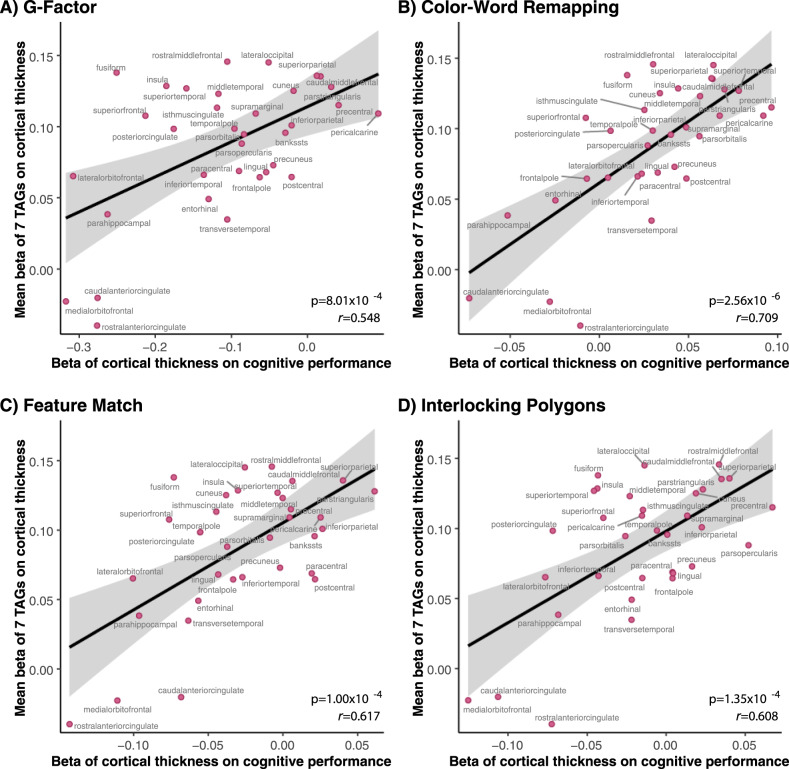
Correlations between interregional variations in associations of seven triacylglycerols with cortical thickness and of cortical thickness with cognitive functioning. To assess these correlations, we used Pearson’s *r*. The figure shows the results for the general cognition “G-Factor”^8^ (**a**) and three most significant individual measures, namely color-word remapping (**b**), feature match (**c**), and interlocking polygons (**d**). The results for the remaining tests are presented in Supplementary Fig. 8.

To examine if our findings have cognitive implications, we tested whether interregional variations in the associations between seven TAGs and cortical thickness (i.e., the “interregional average-beta profile”) correlate with interregional variations in the associations between cortical thickness and measures of cognitive functioning. These analyses showed positive associations for several cognitive measures, as well as for the general cognition “G-Factor”^18^ (*p* = 8.01 × 10^–4^, Fig. 5 and Supplementary Fig. 7). This finding indicates that, in cortical regions where thickness is most closely associated with the seven TAGs, the thickness is also most closely associated with cognitive functioning.

## Discussion

Thickness of the cerebral cortex is related to cognitive functioning in aging adults^19–21^, but the molecular factors modulating this relationship are incompletely understood. We studied associations between 518 circulating TAG species and mean thickness of the cerebral cortex in a general population of middle-aged adults. We identified seven circulating TAG species carrying 18-carbon unsaturated FAs to be positively associated with mean cortical thickness. We also demonstrated that these seven TAG species show larger effect estimates on cortical regions with higher expression of astrocyte- and neuronal cell-specific genes involved in the regulation of long-chain FA metabolism and cellular membranes. Finally, we demonstrate that thickness of cortical regions most closely associated with these seven TAG species is also most closely associated with better cognitive functioning.

Currently, there are no published studies examining associations between thickness of the cerebral cortex and a comprehensive set of circulating TAG species in a general population. A recent study reported associations between mild cognitive impairment (MCI) and Alzheimer’s disease (AD) with serum levels of 84 TAG species in 689 participants from the Alzheimer’s Disease Neuroimaging Initiative (ADNI) cohort (average age 75 years)^22^. The TAG species in that study were defined by the total number of acyl-chain carbons and double bonds, but unlike in the present study, they were not defined by the identity of any of the three esterified FAs. Also unlike in the present study, the primary analyses of that work focused on studying component scores from principal component analysis instead of studying individual TAG species. Supportive of our findings, the authors found that lower component scores of PC5 loaded by PUFA-containing TAGs (PUTGs) were observed in both MCI and AD patients than in healthy controls, and that lower levels of these PUTGs were associated with lower thickness of the entorhinal cortex and hippocampus^22^. The results in our cohort (average age 49 years) indicate that the positive associations between PUTGs and cortical thickness are also seen in generally healthy middle-aged adults, and that the particular PUTG species carrying 18-carbon FAs may play a key role in PUTG associations with thicker cortex.

Circulating total-TAG concentration has been reported as being associated with both thicker^23^ and thinner^24^ cerebral cortex. The former study was completed in a community-based sample of middle-aged adults (average age 51 years), whereas the latter study was carried out in a population (average age 60 years) involving patients with the metabolic syndrome. In the metabolic syndrome, blood levels of saturated FAs are high, whereas levels of PUFAs are low^25^. The contradictory results of the above-described studies could arise from different FA composition of TAGs in the community-based sample of individuals versus patients with the metabolic syndrome. We did not find an association between cortical thickness and total TAG concentration (beta = 0.078; *p* = 0.10). Our main findings, however, are supportive of the possibility that differing FA composition may contribute to the reported variability in the associations of total TAGs with cortical thickness: TAGs carrying long-chain PUFAs rather than TAGs carrying shorter-chain saturated FAs are associated with thicker cerebral cortex.

The seven TAGs we identified as associated with mean cortical thickness carry 18-carbon FAs, but the total circulating concentrations of these FAs were not associated with mean cortical thickness (Supplementary Fig. 3). This finding suggests that brain uptake of essential FAs may be preferred in a form of TAG. A study that reported omega-3 PUFAs esterified to TAGs having superior bioavailability compared with free FAs or FA ethyl esters^26^ provides evidence, at least partially, supporting this hypothesis. Current understanding is that the uptake of FAs to the brain happens via FA-transport proteins or passive diffusion after hydrolysis of TAGs at the BBB:^3^ FA uptake requires concomitant FA activation to acyl-coenzyme A (CoA) by long-chain-fatty-acid-CoA synthases^3^, such as ACSL3^27^, mRNA expression of which was found to be correlated with the TAG-associated effect estimates across the cerebral cortex in the present study. There is, however, evidence that intact TAGs may cross the BBB in mice^28^. It will be an interesting topic for future studies to investigate if intact TAGs can cross the BBB in humans, and if TAG species with a particular FA composition are preferred over others in delivering FAs to the brain.

Five out of the seven TAGs (TAG52:6FA18:3, TAG54:6FA18:3, TAG54:7FA18:3, TAG54:8FA18:3, and TAG56:9FA18:3) we identified as associated with thicker cortex carry FA18:3 that includes the essential ALA. Dietary supplementation of ALA is crucial for normal neural development^29^, and, therefore, ALA is a fundamental constituent of baby formulas^29^. Lack of dietary ALA decreases phospholipid synthesis and induces changes in FA composition of membrane phospholipids in the rat brain^30^. ALA is a precursor for DHA, the key structural FA in the cerebral cortex^3^. The brain’s ability to synthesize DHA from ALA is limited, and thus it is thought that most of the brain DHA is derived from the circulation^31^. Unexpectedly, we did not find significant associations between DHA-containing lipids and cortical thickness. Out of the nominally significant TAG species carrying DHA, TAG56:9FA22:6 showed the strongest association (beta = 0.125, *p* = 0.0074); this association may have been significant in a larger sample. Circulating total-FA22:6 showed a nominally significant (beta = 0.123, *p* = 0.009) association with cortical thickness. In our sample; however, the associations between cortical thickness and FA18:3-containing TAGs were more robust than the associations of total-FA22:6 or FA22:6-containing TAGs. This could imply that, in middle-aged adults, maintenance of higher circulating concentration of ALA-containing TAGs may have more beneficial effect on cortical thickness than maintenance of higher circulating concentration of DHA or DHA-containing TAGs.

The remaining two of the seven TAGs (TAG54:7FA18:1 and TAG54:8FA18:2) associating with thicker cortex in the present study carry FA18:1 and FA18:2. The latter includes essential LA that is used for synthesis of ARA, the other of the predominant FAs in the brain along with DHA^1^. The former includes oleic acid (OA; FA18:1ω9), which is the main monounsaturated FA within the brain^3^ and constitutes ~40% of the FAs in the phospholipid fraction of adult human brain^32^. Considering the essential role of phospholipids in the formation of cellular membranes, it is not surprising that, in the functional element enrichment analyses, we found positive correlations between the seven TAG-associated effect estimates on cortical thickness and expression of neuronal cell-specific genes with function related to cell membranes. Similarly to PUFAs, also OA plays a role in anti-inflammatory signaling in the brain^33^ in addition to its essential role as a structural component in phospholipid bilayers. Overall, a considerable body of literature underscores the multiple functions of FAs in the normal brain physiology. In line with this, recent evidence suggests that perturbations of FA metabolism are closely related to development of neurodegenerative diseases^6,8,34^—the results of the present study may have implications for understanding the role of circulating TAGs in FA delivery into the brain.

This study elucidates the associations between circulating TAGs and the thickness of the human cerebral cortex. Our key finding is that TAG species carrying 18-carbon FAs associate with thicker cortex in middle-aged adults. All significant TAGs had a total of 6 or more double bonds and 52 or more of acyl-chain carbons; overall, the effect sizes of TAGs on cortical thickness were positively correlated with both the number of double bonds and acyl-chain length in TAGs. We found that the interregional variation in TAG-associated effects on cortical thickness was associated with interregional variations of expression of genes specific to astrocytes and pyramidal neurons. Our results further suggest that the seven circulating TAGs may have stronger associations with cortical thickness in regions with higher expression of genes with functions related to cellular membranes and long-chain FA metabolism—here, one of the most intriguing findings was that the effect estimates of the seven TAGs tended to be larger in cortical regions with higher expression of *ACSL3*, a gene encoding an enzyme participating in FA uptake by the brain. Lastly, our findings may have cognitive implications, as thickness of cortical regions most closely associated with the seven TAGs is also most closely associated with better cognitive functioning. Further studies should be designed to determine if dietary interventions can increase circulating TAG-FA18:3 concentrations and if this could help to prevent cognitive impairment related to cortical thinning.

## Methods

### Study population

The Saguenay Youth Study (SYS) is a population-based study conducted in the Saguenay-Lac-St.-Jean region of Quebec, Canada; it is aimed at investigating the etiology and early stages of common cardiometabolic and brain diseases^10^. The SYS includes 486 families with a total of 1028 adolescents and 962 parents^10^. In the present work, we studied data from 441 SYS adults in whom brain magnetic resonance imaging (MRI) and lipidomics data were available, and who were not using lipid-lowering medications. Written consent was obtained from all participants, and the study was approved by local research ethics committees.

### Cortical thickness estimation

T1-weighted (T1W) magnetic resonance imaging (MRI) of the brain was conducted using with a 1.5 Tesla Avanto (Siemens) scanner. The T1W images were acquired using the three-dimensional Magnetization Prepared Rapid Gradient Echo sequence with 176 sagittal slices (1-mm isotropic resolution, TR = 2.400 ms, TE = 2.65 ms, TI = 1000 ms, and flip angle = 8°). FreeSurfer (v 5.3.) cortical reconstruction was used to derive measures of cortical thickness and surface area in 34 regions (per hemisphere), as segmented using the Desikan-Killiany atlas^14^. The mean cortical thickness was calculated as average thickness over the 34 regions and two hemispheres, weighting for cortical area per region.

### Serum lipidomics

All lipidomic measurements were performed using the TrueMass Complex Lipid Panel^TM^ by Metabolon (Morrisville, USA). Lipids were extracted from fasted serum samples and their concentrations (μM) were determined in mass spectrometry analyses.

Majority (78%; *N* = 342) of the study participants had blood withdrawal for lipidomic quantifications and brain MRI scan done on the same day. The average time between blood withdrawal and MRI scan was 0.76 days ± 0.86 (SEM).

### Statistics and reproducibility

#### Linear regression modeling

In the association analyses, mean cortical thickness was modeled as a function of the concentration values of each TAG species in linear regression models. Prior to these analyses, each variable was adjusted for age and sex, and inverse-rank-transformed to normality^11^. The transformed TAG concentrations have mean = 0 and standard deviation (SD) = 1, and thus the effect estimates presented in this paper are in SD-units. Owing to the high correlation of the TAG species concentrations (Supplementary Fig. 8), the number of the independent tests performed is lower than the number of TAGs tested. To estimate the number of independent tests performed, we performed principal component analysis of the TAG data^35,36^. We found that the first nine principal components explained 95% of variation in the TAG data and accordingly we considered statistical significance at *p* < 0.006 (0.05/9), as done previously^11–13,37^.

To study if the TAG associations with mean cortical thickness differ by FA chain length or degree of saturation, we determined the linear fit of the beta estimates derived from each of the 518 linear models and the number of carbons or the number of double bonds in the TAGs.

We performed further supplementary analyses as follows. For insight on whether FAs per se rather than TAGs drive these associations, we determined the associations between mean cortical thickness and (i) circulating concentrations of 21 FA species extracted from TAG fraction of lipids (TAG-FA), and (ii) circulating total concentrations of 28 FA species (total-FA) extracted from all protein or lipid carriers, including TAGs. To evaluate the impact of *APOE* ε4-carrier status on associations between mean cortical thickness and TAG concentrations, we additionally adjusted the linear regression models for *APOE* alleles as determined by rs429358 and rs7412 genotypes. In addition, we tested the associations between total cortical surface area and TAG species in linear models, where the surface area was modeled as a function of the concentration values of each TAG species. In all the supplementary analyses, adjustments and data transformations were the same as those described above.

For the seven TAG species that showed significant associations with mean cortical thickness, we used linear regression models to determine their associations with cortical thickness at each of the 34 cortical regions defined by FreeSurfer (mean of the two hemispheres). As the association profiles across the 34 regions were similar for all seven TAG species (Fig. 3), we derived the beta estimates from these models and calculated their averages in order to determine the “interregional average-beta profile” of the seven TAGs with the thickness at each of the 34 cortical regions (Supplementary Data 2).

#### Virtual histology and functional element enrichment analyses

“Virtual histology” is a method to study relative contributions of nine cell types (CA1 and S1 pyramidal neurons, interneuron, astrocyte, microglia, oligodendrocyte, as well as ependymal, endothelial, and mural cells) to observed differences in cortical thickness across the 34 regions defined by FreeSurfer^15^. To perform virtual histology analyses, the “interregional average-beta profile” of the seven TAGs across the 34 regions was tested for association with interregional profiles of the expression of genes specific to the nine cell types; this was done by comparing distributions of correlation coefficients (*r*) of the interregional average-beta profile with cell-specific gene-expression profiles to the null distribution obtained by shuffling the “cell-type” membership^15,38^.

Within each significant cell type, we tested the correlation coefficients for the “interregional average-beta profile” with interregional profiles of gene expression for all the genes in the cell type. The genes whose expression profiles were positively (*r* > 0) or negatively (*r* < 0) correlated with the “interregional average-beta profile” were tested for enrichment of biological processes or pathways using STRING v11.0^39^.

#### Tests on cognitive performance

The study participants completed a computer-based battery of 12 tests designed to assess executive functioning, reasoning, working memory, and visual-spatial skills (Cambridge Brain Sciences platform, www.cambridgebrainscience.com)^18^. The 12 tests were (1) color-word remapping, (2) spatial planning, (3) self-ordered search, (4) paired associates learning, (5) digit span, (6) spatial span, (7) visuospatial working memory, (8) interlocking polygons, (9) feature match, (10) odd one out, (11) grammatical reasoning, and (12) spatial rotation. In addition, we determined the general factor “g” (G-Factor) representing the correlations in performance scores between all 12 cognitive tasks^18^.

We studied the associations of the performance in each test with thickness of the 34 cortical regions in linear regression models, where each test result or the G-Factor served as outcome and cortical thickness of each region as an explanatory variable. Prior to linear model fitting, all the variables were adjusted for age and sex and transformed using inverse-rank-based normal transformation. We sought to test if this interregional profile of the associations between cortical thickness and cognitive performance is correlated with the interregional profile of the associations between cortical thickness and the “interregional average-beta profile.” To do this, we determined correlation between the effect estimates derived from the two sets of association tests (i.e., the betas of thickness of the 34 cortical regions on cognitive performance vs. the betas of the seven TAGs on thickness of the 34 cortical regions).

### Reporting summary

Further information on research design is available in the Nature Research Reporting Summary linked to this article.

## Supplementary information

Supplementary Figures

Supplementary Data 1

Supplementary Data 2

Supplementary Data 3

Supplementary Data 4

Supplementary Data 5

Supplementary Data 6

Supplementary Data 7

Description of Additional Supplementary Files

Reporting Summary

## Data Availability

The summary statistics are available within the article and its data supplement. The individual level participant data analyzed in this study are available by application from the Saguenay Youth Study.
