# Supplementary material for: Thickness of the cerebral cortex shows positive association with blood levels of triacylglycerols carrying 18-carbon fatty acids
Source: Commun Biol. 2020 Aug 20;3:456. doi: 10.1038/s42003-020-01189-5 (PMC7441395; doi:10.1038/s42003-020-01189-5)

Supplementary Figures

Supplementary Figure 1. Serum concentrations of 518 triacylglycerol species.

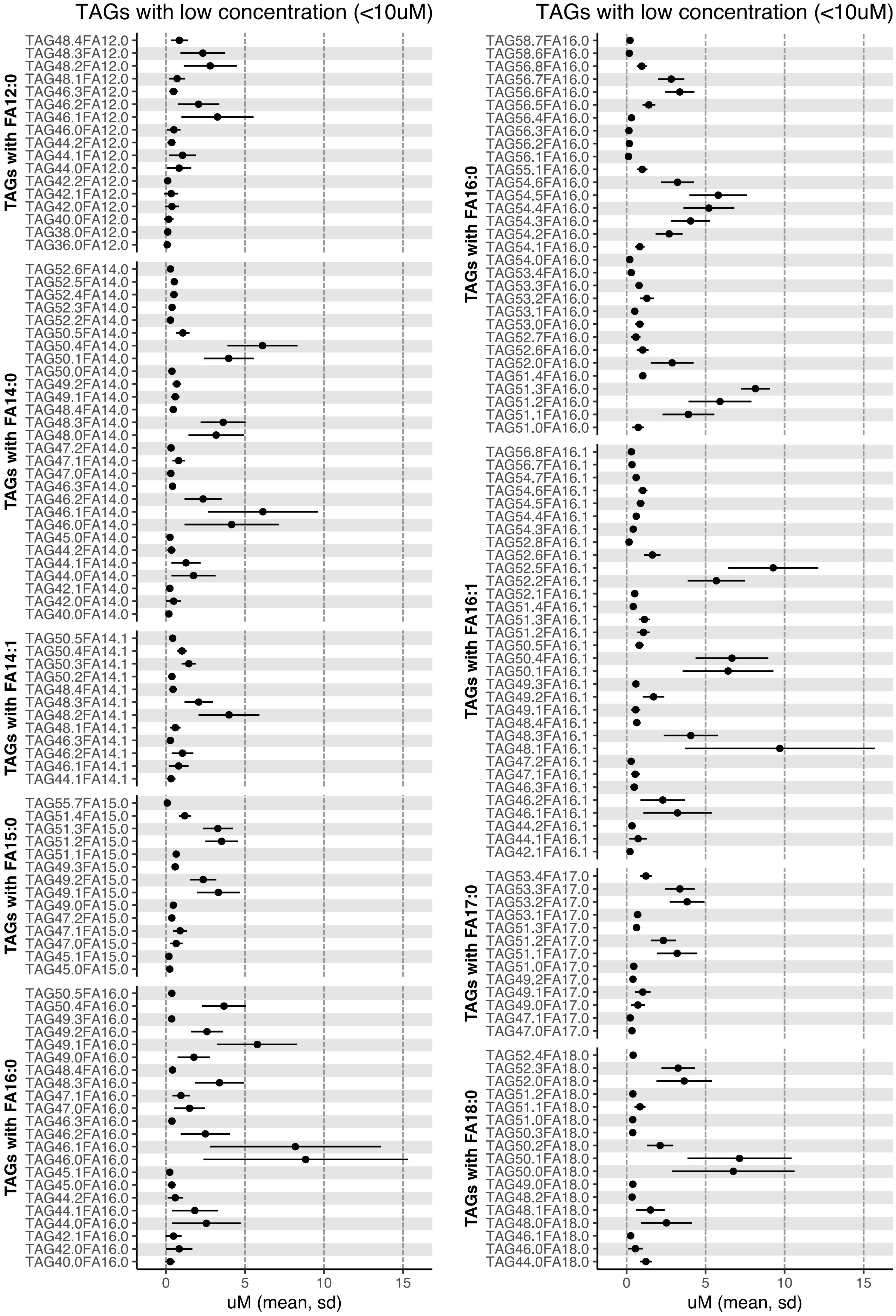

Supplementary Figure 1. (continues)

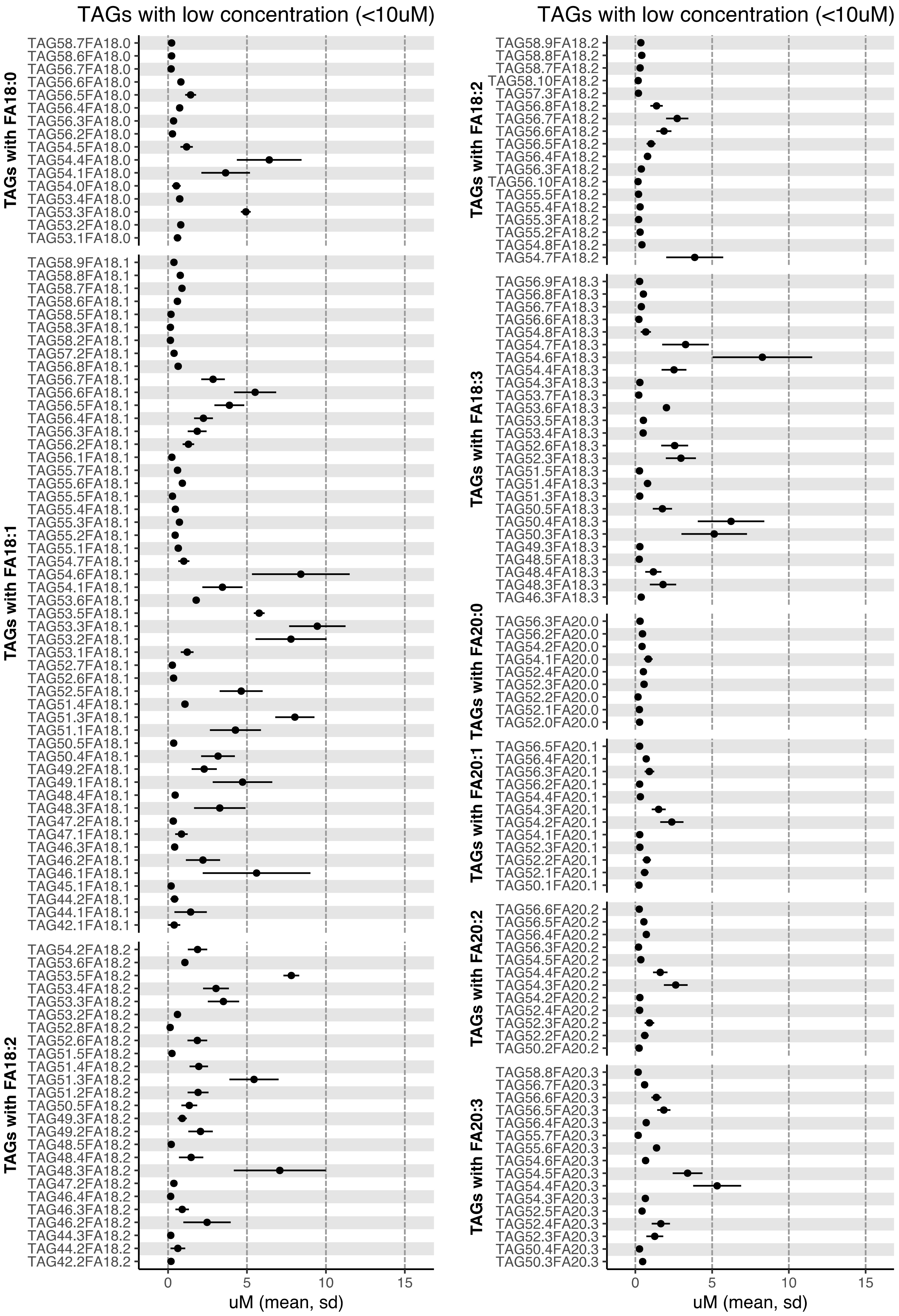

Supplementary Figure 1. (continues)

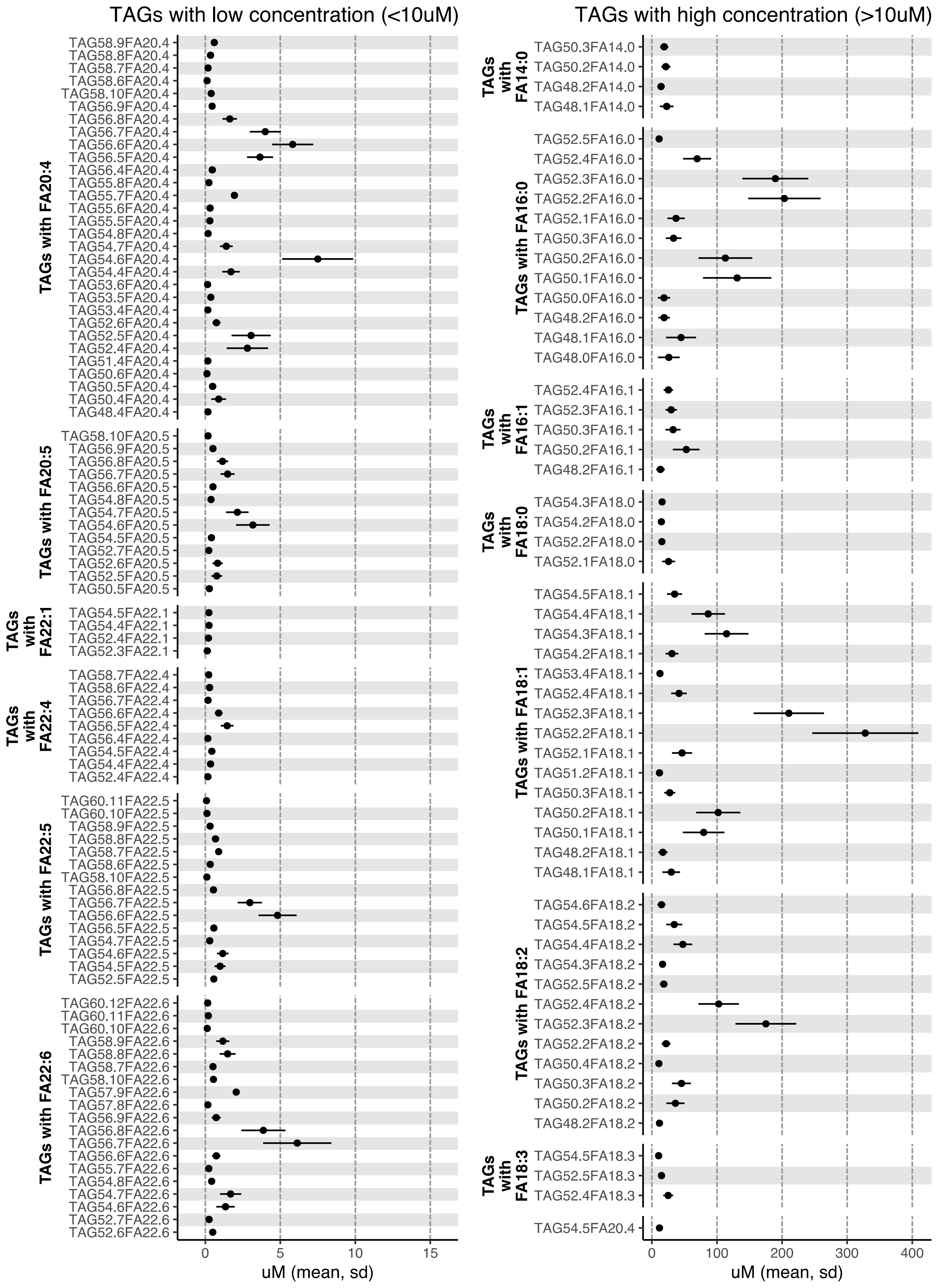

Supplementary Figure 2. Associations of concentrations of fatty acids within triacylglycerols with mean cortical thickness.

Mean thickness of the cerebral cortex was modeled as function of circulating concentration of 21 fatty acid species within triglyceride fraction in linear regression models. Prior to model fitting, each variable was adjusted for age and sex, and inverse rank-transformed to normality.

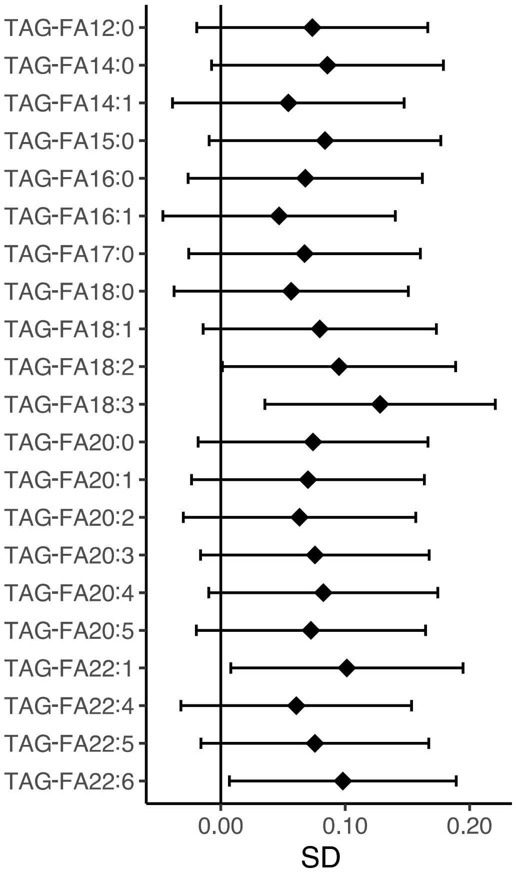

Beta	CI95L	CI95U	P
0.074	-0.019	0.166	0.121
0.086	-0.007	0.179	0.072
0.054	-0.039	0.147	0.254
0.084	-0.009	0.177	0.079
0.068	-0.026	0.162	0.159
0.047	-0.047	0.14	0.327
0.067	-0.026	0.16	0.157
0.057	-0.038	0.151	0.24
0.079	-0.014	0.173	0.097
0.095	0.001	0.189	0.048
0.128	0.035	0.221	0.007
0.074	-0.018	0.166	0.117
0.07	-0.024	0.164	0.143
0.063	-0.03	0.157	0.186
0.076	-0.016	0.167	0.107
0.082	-0.01	0.174	0.081
0.072	-0.02	0.164	0.124
0.101	0.008	0.195	0.034
0.061	-0.032	0.153	0.201
0.076	-0.016	0.167	0.107
0.098	0.007	0.189	0.036

Supplementary Figure 3. Associations of total concentrations of fatty acids with mean cortical thickness.

Mean cortical thickness was modeled as function of total circulating concentration of 28 fatty acid species in linear regression models. Prior to model fitting, each variable was adjusted for age and sex, and inverse rank-transformed to normality.

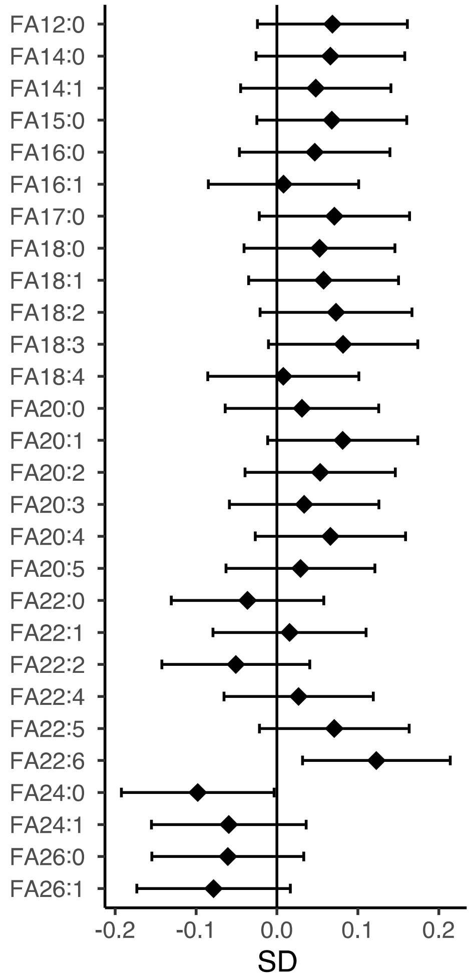

Beta	CI95L	CI95U	P
0.069	-0.024	0.161	0.148
0.066	-0.026	0.158	0.16
0.048	-0.045	0.141	0.313
0.068	-0.025	0.16	0.152
0.047	-0.046	0.14	0.326
0.008	-0.085	0.101	0.865
0.071	-0.022	0.164	0.135
0.053	-0.041	0.146	0.27
0.058	-0.035	0.15	0.224
0.073	-0.021	0.167	0.129
0.082	-0.011	0.174	0.083
0.008	-0.086	0.101	0.87
0.031	-0.064	0.126	0.524
0.081	-0.012	0.174	0.087
0.053	-0.04	0.146	0.261
0.034	-0.059	0.126	0.477
0.066	-0.027	0.159	0.164
0.029	-0.063	0.121	0.536
-0.036	-0.131	0.058	0.449
0.016	-0.079	0.11	0.748
-0.051	-0.142	0.041	0.276
0.027	-0.066	0.119	0.571
0.071	-0.022	0.163	0.134
0.123	0.032	0.214	0.009
-0.098	-0.192	-0.004	0.043
-0.06	-0.155	0.036	0.222
-0.061	-0.155	0.033	0.206
-0.078	-0.173	0.016	0.106

Supplementary Figure 4. Comparisons of the effect estimates and corresponding 95% confidence intervals obtained in analyses adjusted for age and sex with the results obtained in analyses adjusted additionally for *APOE* alleles.

Results of the primary analyses were compared with results of linear regression models, where mean cortical thickness was modeled as function of each TAG species concentration, and adjusted for age, sex, and additionally also for *APOE* alleles determined by rs429358 and rs7412 genotypes.

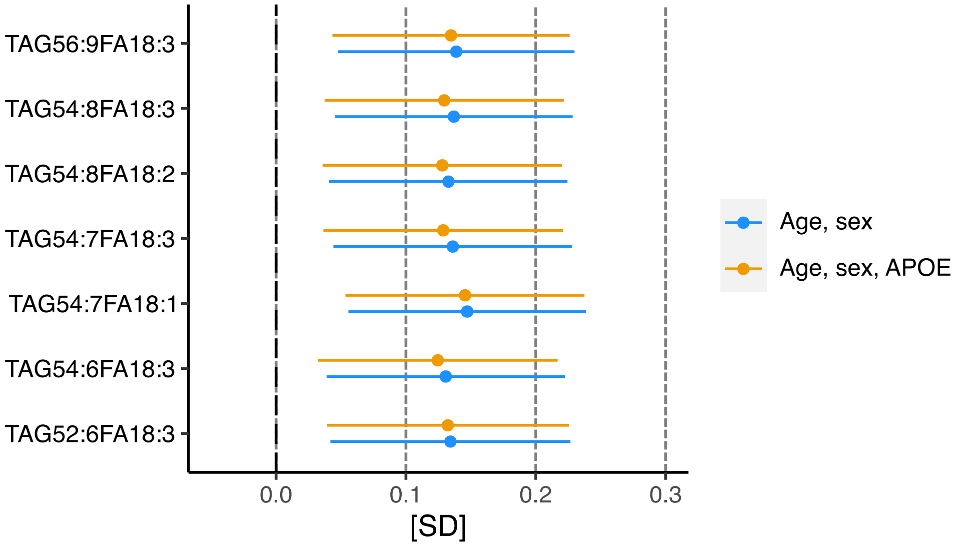

Supplementary Figure 5. Associations of serum concentrations of 518 TAGs with total cortical area.

Total cortical surface area was modeled as function of each TAG species concentration in linear regression models. Prior to model fitting, each variable was adjusted for age and sex, and inverse rank-transformed to normality.

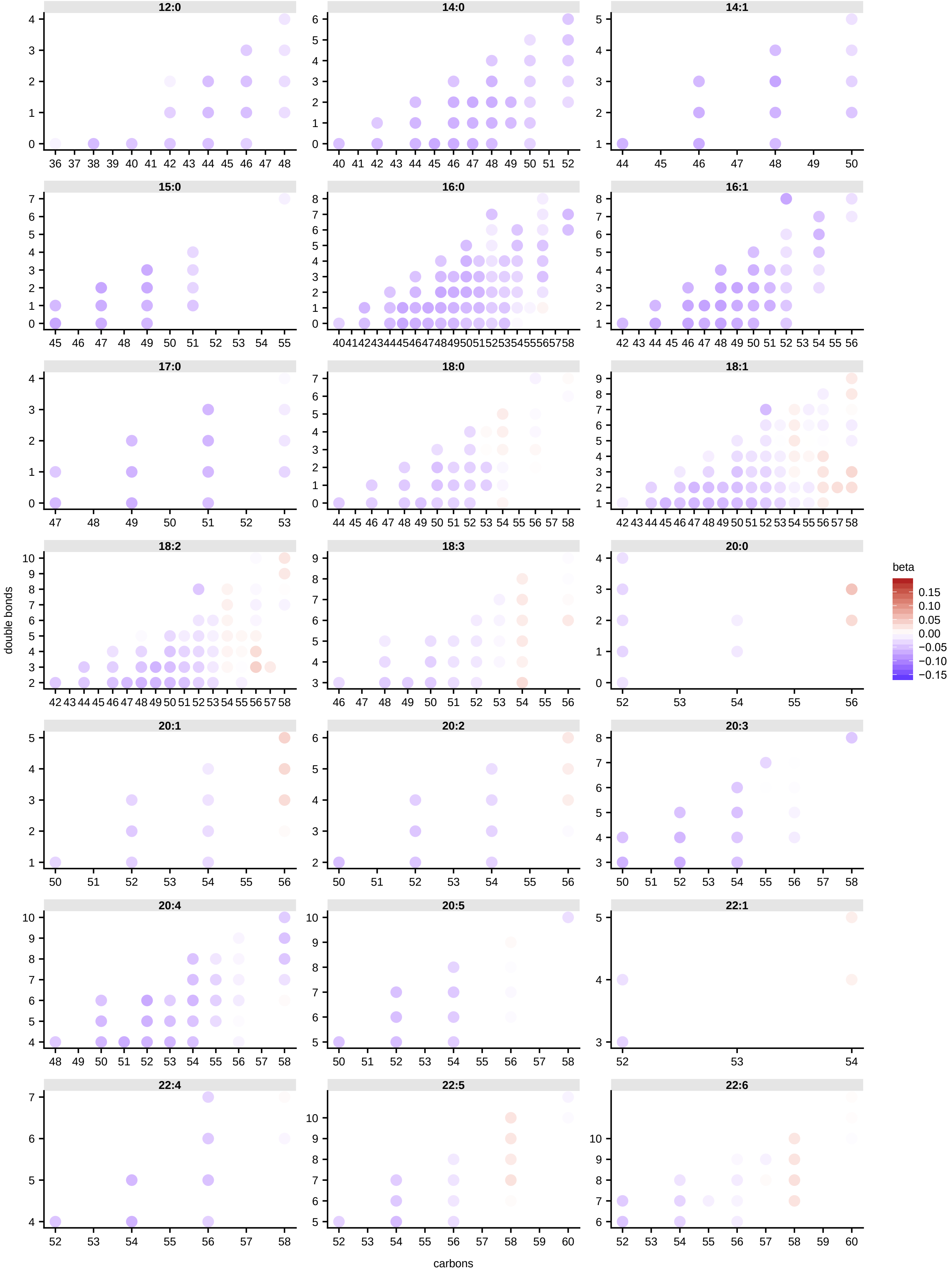

Supplementary Figure 6. Results from virtual histology for the interregional 'average beta profile' of the 7 triacylglycerols positively associated with mean cortical thickness.

Distributions of the correlation coefficients between the interregional 'average beta profile' and cell-type-specific gene expression profiles. Each panel shows the estimated probability density (y-axis) for the correlation coefficients (x-axis) for the 'average beta profile' with the expression profiles of the genes that belong to the given cell type. Gray box represents 2.5% and 97.5% of critical values obtained from the empirical null distribution of the correlation coefficients within each cell type. The dashed line indicates the average correlation coefficient. Asterisk(*) indicates cell types that have correlation coefficient distributions different from the empirical null distributions at the significance level *p*<0.05.

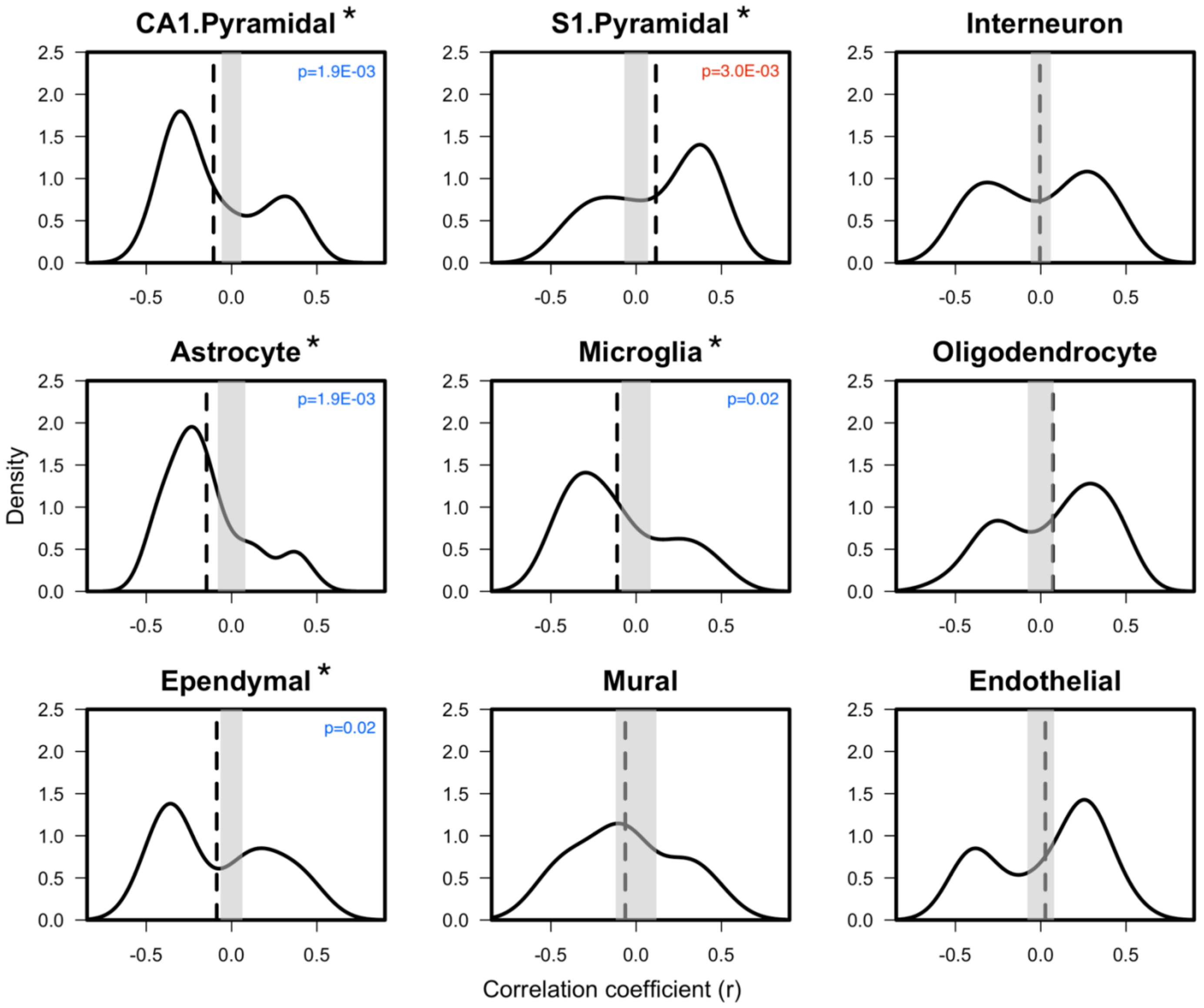

Supplementary Figure 7. Corresnpondence of the association profiles of triacylglycerols and cognitive performance with cortical thickness.

The associations between thickness of the 34 cortical regions and cognitive performance (12 tests and the “G-Factor” describing the performance in all the tasks) were determined in linear regression models. Each test result served as outcome and thickness of each cortical region as an explanatory variable. Prior to linear model fitting, all the variables were adjusted for age and sex, and inverse rank-transformed to normality. The linear fit of the effect estimates derived from these models (x-axes) and the ‘average beta profile’ of the 7 TAGs (described previously; y-axes) was tested to study if cortical regions with thickness most closely associated with cognitive performance are also the regions with thickness most closely associated with the TAGs. Each data point denotes one of the 34 cortical regions.

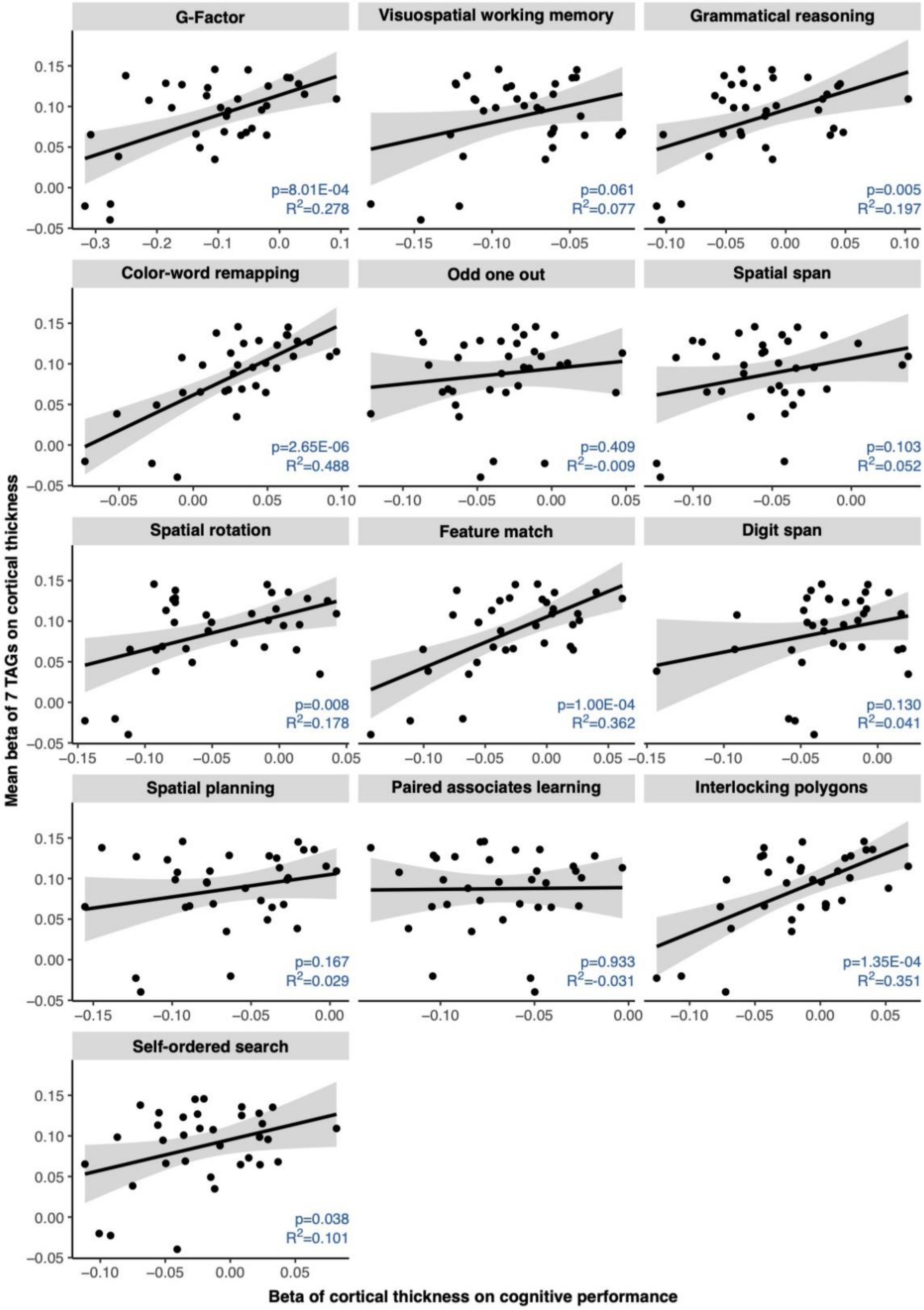

Supplementary Figure 8. Dimensions 1 and 2 from the principal component analysis of the concentrations of 518 triacylglycerol species.

The 7 triacylglycerol species showing significant association with the mean thickness of the cerebral cortex in the present study are highlighted with red color.

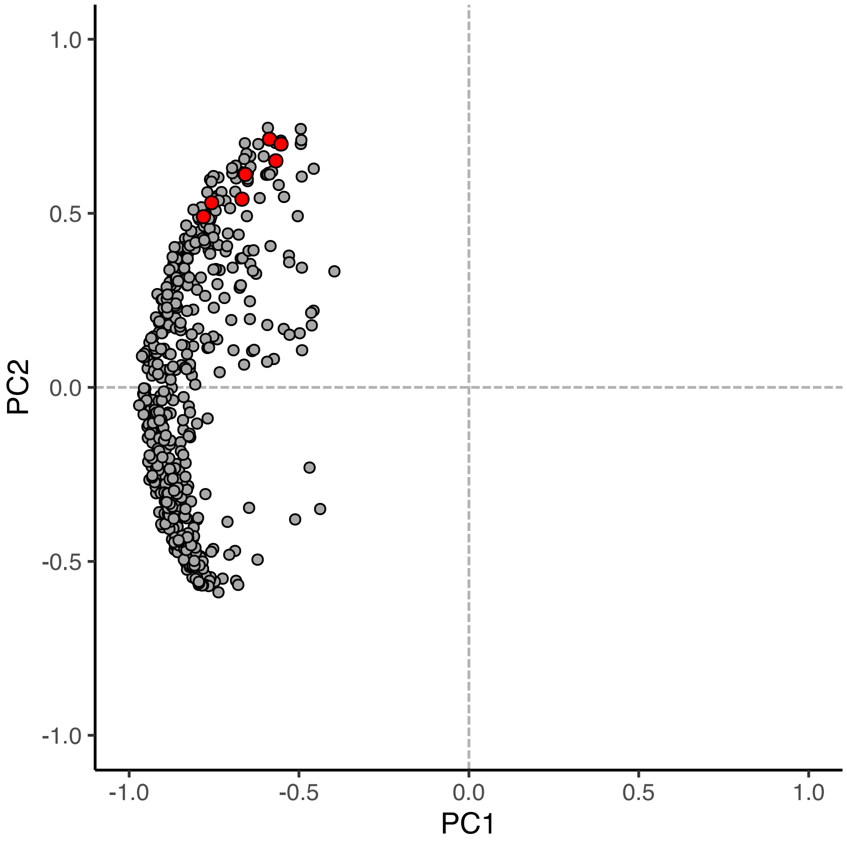

Supplement: Supplementary file 1 — Supplementary Figures [file 42003_2020_1189_MOESM1_ESM.pdf]
