# Supplementary material for: Thickness of the cerebral cortex shows positive association with blood levels of triacylglycerols carrying 18-carbon fatty acids
Source: Commun Biol. 2020 Aug 20;3:456. doi: 10.1038/s42003-020-01189-5 (PMC7441395; doi:10.1038/s42003-020-01189-5)
Supplement: Supplementary file 9 — Description of Additional Supplementary Files [file 42003_2020_1189_MOESM9_ESM.pdf]

Description of Additional Supplementary Files

Supplementary Data 1. Triacylglycerol concentrations and associations with mean cortical thickness

Supplementary Data 2. Beta estimates of the 7 TAGs on the thickness of 34 cortical regions and their averages (average beta profile)

Supplementary Data 3. Functional enrichment of astrocyte-specific genes expression of which is associated with the 'average beta profile'

Supplementary Data 4. Functional enrichment of CA1 pyramidal cell-specific genes expression of which is associated with the 'average beta profile'

Supplementary Data 5. Functional enrichment of S1 pyramidal cell-specific genes expression of which is associated with the 'average beta profile'

Supplementary Data 6. Functional enrichment of ependymal cell-specific genes expression of which is associated with the 'average beta profile'

Supplementary Data 7. Functional enrichment of microglia-specific genes expression of which is associated with the 'average beta profile'
