# Supplementary material for: Thickness of the cerebral cortex shows positive association with blood levels of triacylglycerols carrying 18-carbon fatty acids
Source: Commun Biol. 2020 Aug 20;3:456. doi: 10.1038/s42003-020-01189-5 (PMC7441395; doi:10.1038/s42003-020-01189-5)
Supplement: Supplementary file 10 — Reporting Summary [file 42003_2020_1189_MOESM10_ESM.pdf]

Corresponding author(s): Zdenka Pausova

Last updated by author(s): Jul 16, 2020

Reporting Summary

Nature Research wishes to improve the reproducibility of the work that we publish. This form provides structure for consistency and transparency in reporting. For further information on Nature Research policies, see our Editorial Policies and the Editorial Policy Checklist.

Statistics

For all statistical analyses, confirm that the following items are present in the figure legend, table legend, main text, or Methods section.

n/a	Confirmed	
☐	☒	The exact sample size (*n*) for each experimental group/condition, given as a discrete number and unit of measurement
☐	☒	A statement on whether measurements were taken from distinct samples or whether the same sample was measured repeatedly
☐	☒	The statistical test(s) used AND whether they are one- or two-sided<br>*Only common tests should be described solely by name; describe more complex techniques in the Methods section.*
☐	☒	A description of all covariates tested
☐	☒	A description of any assumptions or corrections, such as tests of normality and adjustment for multiple comparisons
☐	☒	A full description of the statistical parameters including central tendency (e.g. means) or other basic estimates (e.g. regression coefficient) AND variation (e.g. standard deviation) or associated estimates of uncertainty (e.g. confidence intervals)
☐	☒	For null hypothesis testing, the test statistic (e.g. F, t, r) with confidence intervals, effect sizes, degrees of freedom and P value noted<br>*Give P values as exact values whenever suitable.*
☒	☐	For Bayesian analysis, information on the choice of priors and Markov chain Monte Carlo settings
☒	☐	For hierarchical and complex designs, identification of the appropriate level for tests and full reporting of outcomes
☐	☒	Estimates of effect sizes (e.g. Cohen's d, Pearson's r), indicating how they were calculated

Our web collection on statistics for biologists contains articles on many of the points above.

Software and code

Policy information about availability of computer code

Data collection	FreeSurfer (v 5.3.) cortical reconstruction was used to derive measures of cortical thickness and surface area.
Data analysis	All analyses were performed in R version 3.6.2.

For manuscripts utilizing custom algorithms or software that are central to the research but not yet described in published literature, software must be made available to editors and reviewers. We strongly encourage code deposition in a community repository (e.g. GitHub). See the Nature Research guidelines for submitting code & software for further information.

Data

Policy information about availability of data

All manuscripts must include a data availability statement. This statement should provide the following information, where applicable:

- Accession codes, unique identifiers, or web links for publicly available datasets
- A list of figures that have associated raw data
- A description of any restrictions on data availability

The summary statistics are available within the article and its data supplement. The individual level participant data analyzed in this study are available by application from the Saguenay Youth Study.

Field-specific reporting

Please select the one below that is the best fit for your research. If you are not sure, read the appropriate sections before making your selection.

☒ Life sciences ☐ Behavioural & social sciences ☐ Ecological, evolutionary & environmental sciences

For a reference copy of the document with all sections, see nature.com/documents/nr-reporting-summary-flat.pdf

Life sciences study design

All studies must disclose on these points even when the disclosure is negative.

Sample size	We used the maximal sample size (individuals with full data) available in our cohort, Saguenay Youth Study.
Data exclusions	As lipid-lowering medications have robust effect on circulating triglyceride levels, we excluded individuals using any lipid-lowering medications.
Replication	We have not found a collaborator with suitable brain MRI data and blood triglycerides quantified with Metabolon's Complex Lipid platform - thus, we have not been able to replicate our findings.
Randomization	We don't have multiple experimental groups, and thus we have not performed randomization. We have adjusted our analyses for age and sex, and in addition for APOE alleles, as these all are potential confounding factors influencing both lipid and brain phenotypes.
Blinding	We don't have multiple experimental groups nor randomization, and therefore blinding is not relevant in our study.

Reporting for specific materials, systems and methods

We require information from authors about some types of materials, experimental systems and methods used in many studies. Here, indicate whether each material, system or method listed is relevant to your study. If you are not sure if a list item applies to your research, read the appropriate section before selecting a response.

Materials & experimental systems

n/a	Involved in the study	
☒	☐	Antibodies
☒	☐	Eukaryotic cell lines
☒	☐	Palaeontology and archaeology
☒	☐	Animals and other organisms
☐	☒	Human research participants
☒	☐	Clinical data
☒	☐	Dual use research of concern

Methods

n/a	Involved in the study	
☒	☐	ChIP-seq
☒	☐	Flow cytometry
☐	☒	MRI-based neuroimaging

Human research participants

Policy information about studies involving human research participants

Population characteristics	Our study population consisted of 441 middle-aged adults from the Saguenay Youth Study (males 43%, average age 49 years, mean BMI 27.6). Study participants are generally healthy. Individuals on lipid-lowering medications were excluded.
Recruitment	The Saguenay Youth Study is a family-based study, including 1029 adolescents and their 962 parents. The study was recruited via adolescents attending high schools in the Saguenay-Lac-Saint-Jean region of Quebec, Canada. Both maternal and paternal grandparents of the adolescents were required to be of French-Canadian ancestry and born in the region; as such, all adolescents and their parents are of a single ethnicity (European ancestry).
Ethics oversight	The study was approved by the research ethics committees of Chicoutimi Hospital (Chicoutimi, QC, Canada) and the Hospital for Sick Children (Toronto, ON, Canada).

Note that full information on the approval of the study protocol must also be provided in the manuscript.

Magnetic resonance imaging

Experimental design

Design type	N/A

Design specifications: N/A

Behavioral performance measures: N/A

Acquisition

Imaging type(s): Structural T1-weighted images

Field strength: 1.5T

Sequence & imaging parameters: 3D Magnetization Prepared Rapid Gradient Echo sequence with 176 sagittal slices (1-mm isotropic resolution, TR = 2.400ms, TE = 2.65ms, TI = 1000ms, and flip angle = 8°).

Area of acquisition: Whole brain

Diffusion MRI: ☐ Used ☒ Not used

Preprocessing

Preprocessing software: T1W images processed with FreeSurfer, version 5.3

Normalization: FreeSurfer, version 5.3

Normalization template: FreeSurfer, version 5.3

Noise and artifact removal: N/A

Volume censoring: N/A

Statistical modeling & inference

Model type and settings: N/A

Effect(s) tested: N/A

Specify type of analysis: ☐ Whole brain ☒ ROI-based ☐ Both

Anatomical location(s): 34 regions (per hemisphere), as segmented using the Desikan-Killiany atlas

Statistic type for inference (See Eklund et al. 2016): N/A

Correction: N/A

Models & analysis

n/a	Involved in the study
☒	☐ Functional and/or effective connectivity
☒	☐ Graph analysis
☒	☐ Multivariate modeling or predictive analysis
